# Telomeres and Telomerase in Heart Ontogenesis, Aging and Regeneration

**DOI:** 10.3390/cells9020503

**Published:** 2020-02-22

**Authors:** Denis Nalobin, Svetlana Alipkina, Anna Gaidamaka, Alexander Glukhov, Zaza Khuchua

**Affiliations:** 1 Faculty of Biology, Lomonosov Moscow State University, 119991 Moscow, Russian; 2 Department of Biochemistry, Sechenov First Moscow State Medical University, 119991 Moscow, Russian; 3 Institute of Chemical Biology Ilia State University, 0162 Tbilisi, Georgia; 4 Division of Molecular and Cardiovascular Biology, Cincinnati Children’s Medical Center, Cincinnati, OH 45229, USA

**Keywords:** cardiomyocytes, telomere length, telomerase activity, development, regeneration, reactive oxygen species

## Abstract

The main purpose of the review article is to assess the contributions of telomere length and telomerase activity to the cardiac function at different stages of development and clarify their role in cardiac disorders. It has been shown that the telomerase complex and telomeres are of great importance in many periods of ontogenesis due to the regulation of the proliferative capacity of heart cells. The review article also discusses the problems of heart regeneration and the identification of possible causes of dysfunction of telomeres and telomerase.

## 1. Introduction

The cardiovascular system plays a vital for the whole organism. It performs many vital functions such as supplying organs and tissues with nutrients, hormones, carrying oxygen to the cells and maintaining physiological temperature.

According to the World Health Organization, diseases of the cardiovascular system are the main cause of lethality worldwide. Every year, more than 7.4 million people die from coronary heart disease worldwide, and these rates continue to grow. Congenital heart defects are also highly prevalent and may be a cause of serious complications in the future. Thus, the search for solutions to the cause and consequences of heart diseases is one of the most serious biomedical problems.

The possibility to regenerate the mammalian heart is still rarely studied; however, studying it would allow finding new ways to restore the heart after severe injuries.

A huge number of factors affect the functioning of the heart. One of them is cell aging, which manifests itself in the form of various disorders. Cellular aging is associated to a greater extent with the loss of telomere length and a decrease in telomerase activity.

Understanding the processes of regulation of telomere length and telomerase activity in the ontogenesis of cardiac tissue can help to understand the causes of heart disease in one or another period of development of the organism.

## 2. Telomeres and Telomere Complex

In 1961, Leonard Hayflick discovered the limit of somatic cell division in vitro. The limit is named after the scientist—the Hayflick limit. According to Hayflick’s observations, human fibroblasts dividing in a cell culture died after approximately 50 divisions. The cells showed signs of aging, stopped dividing and underwent programmed cell death [1].

**Figure 1:**
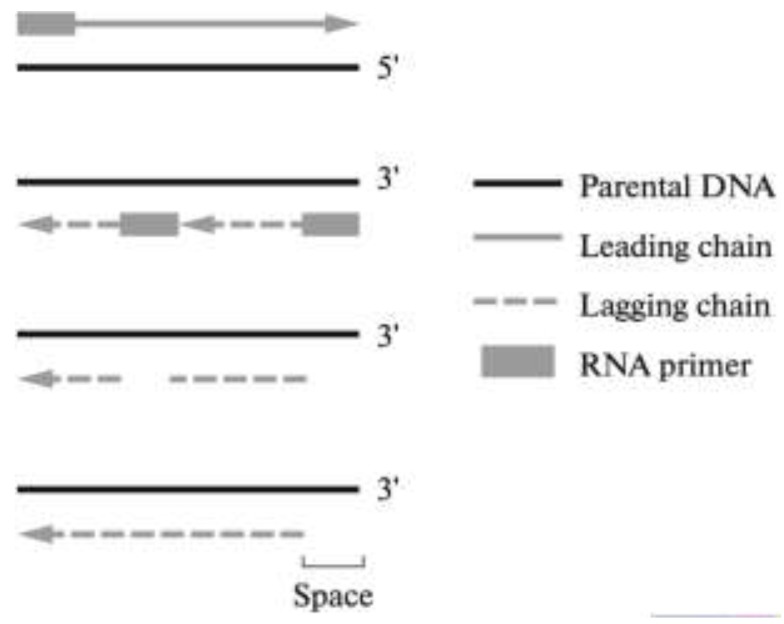
Problems associated with chromosomes’ terminal under-replication [2].

Ten years after the discovery of the Hayflick limit, Olovnikov hypothesized that DNA polymerase cannot copy a small region at the end of chromosome (telomere), which leads to terminal under-replication of DNA [2] (Figure 1).

Scientists postulated that telomeres undergo shortening with each cell division and assumed that this phenomenon was associated with the cell division limit. This hypothesis was confirmed in 1985, when Greider and Blackburn (1985) identified an enzyme in the ciliate *Tetrahymena thermophila* that prevented the degradation of the ends of chromosomes. The enzyme was named telomerase [2].

Vertebrate telomeres consist of repeating TTAGGG sequences at the ends of chromosomes and maintain their integrity. Since DNA replication is asymmetric at both strands, the sequence at the 3′ end would lose 30–200 nucleotides with each cycle of DNA replication and cell division. Telomeres have non-coding recurring sequences at the 3′ end to prevent the loss of coding sequences during replication [3]. Moreover, telomeres are covered with Shelterin complex consisting of six proteins: TRF1 (telomere repeat binding factor 1), TRF2 (telomere repeat binding factor 2), TIN2 (TRF1-interacted nuclear protein 2), RAP1 (rif-associated protein), POT1 (protection of telomeres) and TPP1 (telomere protected protein 1). Telomeres end with a single-stranded 3′-end, which has a compact T-loop structure that maintains their stability [4]. Telomeres were proposed as mitotic clocks that show how many times a cell has divided [5].

When telomeres shorten to a critical length, the cell goes into a state of senescence, which initiates a series of changes in gene expression patterns of cell cycle inhibitors, decreases cellular proliferative potential and activates apoptosis [6].

Telomerase is responsible for telomere elongation and consists of an RNA component (TERC) and telomerase reverse transcriptase (TERT), a catalytic component. TERT uses TERC as a template for synthesizing new repeats of telomeric DNA at the single-stranded ends of chromosomes [7]. Most somatic cells lack telomerase activity, but undifferentiated germ cells, stem cells, activated lymphocytes and most tumor cells have a high level of telomerase activity to overcome telomere contraction and maintain limitless cell division. However, differentiated resting cells usually have a low or undetectable level of telomerase activity [8].

## 3. Embryonic Development of the Heart

The heart begins to function in the early stages of development in both mammals and lower vertebrates such as *Danio rerio* (zebrafish) [9,10]. In mice, the level of proliferation of cardiomyocytes (CM) is high in early embryogenesis, and then it gradually decreases until the 10th to 12th day of embryonic development (E10–12) when the heart is almost fully formed [9,11]. Similar dynamics are also shown for telomerase: its activity is detected in the heart tissue of the human fetus until the 12th week of embryonic development, which coincides with the histological differentiation of the myoblasts of the heart into cardiomyocytes [12]. This observation is consistent with the fact that, by the sixth month of prenatal development, the morphological appearance of the heart muscle is almost the same as that of an adult [12].

However, a full picture of dynamics of telomerase activity during the cardiac embryonic development is still unclear. It is known that activity is registered during E11.5 [13] and E16.5 in mice [14], as well as on E10 and E20 in rats. Moreover, telomerase activities in developing rat hearts start to decline after E10 [15]. Dynamics of telomerase inactivation in developing hearts of rats and humans appear to have similar patterns since, in rats, the heart becomes a fully formed functional organ by E16 [16].

## 4. Early Postnatal Heart Development

Proliferation reaches the first minimum point in the heart of newborn mice (i.e., day 0 of postnatal development; P0) [17]. During this period, the system that is responsible for the cell cycle is transformed from embryonic to postnatal mode. Before birth, the number of CMs increases, and after birth, it remains almost unchanged. At the same period, tetraploid and binuclear CMs begin to appear [17]. At P3, the peak of mitotic activity appears again, which correlates with an increased number of binuclear CMs (up to 80%) and a decrease in the number of mononuclear CMs. At the same time, both in binuclear and mononuclear CM populations, there is a transition to the G1 phase and cessation of the cell cycle [17]. After P3 there is a sharp decrease in the number of CMs that have entered mitosis [17,18].

If we take a look at the activity of telomerase in the heart at this stage of development, we find a correlation both with a decrease of proliferation and with the advent of binuclear and polyploid CMs. Therefore, it can be speculated that negative telomerase regulation may be important for permanent stopping of the CM cell cycle [15]. Thus, in newborn mice, gradual suppression of telomerase activity occurs, and by P2 the activity decreases by more than 65% [14]. By the third month of postnatal development, only a very small number of *Tert*-expressing cells remain [19]. A number of studies have shown a sharp decline in telomerase activity in newborn mice relative to the hearts of E11.5, and after P10 it is almost undetectable [13]. *Tert* expression has a similar dynamic, which indicates a possible mechanism for the suppression of telomerase activity through the catalytic subunit of telomerase [13,20].

Similar to previous data, it was found that five days after birth, the activity of telomerase in the rat heart was only 20% of the activity at E10. Telomerase activity was absent in P20 heart and remained below the detection limit up to four months of age [15].

Regarding the distribution of telomerase activity in the heart, *TERT* expression is found in a population of cells, including CM, fibroblasts and endothelial cells [19].

The decrease in proliferation potential of CMs positively correlates with telomere depletion in newborn mice. Rapid reduction of telomere length occurs within the first two weeks after birth. Further, the length of the telomeres does not change, as the CMs leave the cell cycle, which contributes to the conservation of telomere reserves. In the first days after birth, the proliferating CMs have a longer telomere than non-proliferating CMs. However, after P15, these differences are already nullified [13].

There are several possible causes for the sharp drop in telomere length in newborn mice. Telomeres shorten during phase S due to the inability of the DNA replication mechanism to support the ends of linear DNA molecules [5]. Therefore, the absence of telomerase predetermines the loss of telomere reserves in CM during the period of their postnatal DNA replication.

More surprising is the high rate of loss of telomeres, starting with P1, which leads to a significant increase in DNA damage in telomeric sites in a one-week period [13]. In this regard, another cause of damage and further loss of telomeres may be the appearance of reactive oxygen species (ROS) in CM after the metabolic transition from anaerobic glycolysis to mitochondrial oxidative phosphorylation during the first week after birth. So, it was found that the level of ROS increased in newborn mice, which probably leads to an increase in the number of DNA damage foci (replacement of guanine with 8-oxo-7,8-dihydroguanine) between P4 and P7 [21]. Such alterations have a particularly noticeable effect on the promoter regions of genes that have a high GC content [22]. Telomeres also have a high GGG content, which makes them an ideal target for ROS attacks [23]. Due to an increase in the level of oxidizers and disturbances in telomeres, the DNA damage response is activated, which leads to a halt in proliferation through activation of the cell cycle inhibitor p21 [13,21]. However, if the oxygen concentration or the ROS levels are reduced, then the proliferation window of the CMs in the early postembryonic period expands along with the increase in the numbers of mononuclear cells relative to two- and multi-nuclei CMs [21]. ROS can be associated with telomerase inhibition due to telomere DNA damage or deoxynucleotides oxidation that explains a sharp drop of telomerase activity after birth [24].

The mechanism that promotes cell cycle arrest in postnatal CMs can also be associated with the gap-fusion-bridge cycle, which leads to tetraploidization, appearance of binucleated cells and inhibition of proliferation [25]. In the gap-fusion-bridge cycle, chromosomes with nonfunctional telomeres merge with each other, forming bridges during mitosis. These chromosome bridges may eventually collapse under the action of forces emanating from the anaphase poles, and further proliferation is inhibited. In murine CMs, decrease in telomeres is associated with the appearance of chromosomal bridges between the daughter nuclei: eight days after birth, CMs display the presence of chromosomal bridges, which correlates with a decrease in telomere length. A potential genomic imbalance caused by the breakdown of chromosomal bridges in binucleated CMs can be a barrier to proliferation [13].

The knockout for the *Terc* gene confirms the role of telomeres in proliferation. Third-generation *Terc*-null mice have shorter telomeres, anaphase bridges and a lower proliferation level than wild-types (WTs) at P1 [13]. 

It should be noted that in lower vertebrates, such as *D. rerio*, telomerase activity in many tissues is sufficiently high both during embryonic and postnatal development [26]. This observation is used to study the proliferative potential of CMs in the regenerative process [27]. In addition, it was shown that CMs of *D. rerio* are single-nucleated and diploid [28,29], which may be associated with high telomerase activity capable of maintaining sufficient telomere length for normal proliferation [28,29].

## 5. Prepubertal Period

CMs of mammals lose their ability to proliferate after birth due to telomere dysfunction and reduced telomerase activity [13]. However, there is evidence of a proliferation surge in the prepubertal period. Thus, from P14 to P15, activation of mitosis with subsequent cytokinesis is observed in both mononucleated or binucleated CMs. Proliferation is accompanied by the expression of cell cycle regulating genes [30]. From this, it can be speculated that there is a molecular mechanism for overcoming the proliferative barrier associated with short telomeres and low telomerase activity. The authors of the study suggest that the wave of synthesis of the hormone triiodothyronine is an impulse for the induction of mitosis [30]. On the other hand, the cause of the activation of proliferation can be telomere elongation due to the start of telomerase expression. This hypothesis is derived from data on increasing the length of telomeres and subsequently the level of proliferation due to the introduction of Tert in the heart of an adult mouse in response to heart damage [20]. At the same time, there is another point of view on this observation: during growth, the size of the heart increases almost exclusively due to hypertrophy, but not hyperplasia. It was shown that the increase in the number of CMs or proliferation rate was not observed between P13 and P100, and that no active DNA synthesis occurred [31]. Similar results were demonstrated for P14–P21 [32].

However, the contribution of hyperplasia to an increase in the size of the heart should not be completely ruled out, although it is probably not as high as was presented in the study of Naqvi et al., 2014 [30]. Indeed, it has been found that proliferating mononuclear cells with increased telomerase activity are present in the hearts of young cats, and they have the physiological properties of immature cells in the form of calcium current in T-type channels [33]. Mitotic activity in human mononuclear CMs is shown during the first 20 years of life (1.9% of the total number of CM), which decreases, but is registered up to the age of 40 [34], as confirmed by earlier works [35]. There is also evidence of an increase in ploidy of CMs and the absence of growth in the number of binuclear cells during the first 20 years of life [35].

## 6. Heart of Adult Vertebrates 

As described above, telomerase activity in mammals drops to a minimum shortly after birth, which causes telomere shortening. However, in the heart of adult animals, as shown in mice, telomerase activity is registered, both in CMs, and in fibroblasts and endothelial cells [19].

CMs of adult mice vary in size: binucleated and multinucleated CMs undergo hypertrophy, and small mononucleated CMs show features of proliferating cells. The length of telomeres in these classes of CMs is inversely related to cell size. In addition, *p16^CDKN2^* expression is observed in large binucleated and multinucleated CMs with the shortest telomeres [36]. P16^CDKN2^ specifically binds and inhibits cyclin-dependent kinases CDK4 and CDK6, which act as regulators of the progression of the cell cycle in G1, contribute to the phosphorylation of the retinoblastoma protein (pRB) and induce cell cycle arrest [37].

With a TRF assay, it has been demonstrated that, unlike mammals, telomerase activity in the hearts of *D. rerio* is high throughout life, and telomere length remains almost unchanged [38]. Further studies were conducted on changes in telomere length in fish of different ages using the Q-Fish method. It was shown that in *D. rerio* the activity of telomerase and the length of telomeres in CMs also varied with age, and the aging of fish leads to a decrease of telomere length and telomerase activity [26].

## 7. Heart Aging

Aging of the heart includes a number of physiological changes that increase the risk of developing diseases and conditions that are hazardous to health. Cardiac diseases are associated with age and have a detrimental impact on the whole organism. However, it remains unclear how cellular aging of heart tissue affects the appearance of heart diseases.

It is known that telomere length decreases with age. For human heart tissue, telomere loss is approximately 20 base pairs per year [39]. In addition to this, it was found that in old rats there was a tendency to reduce the length of terminal restriction fragments (TRF) related to the telomeric and subtelomeric region. Interestingly, only the heart showed a significant decrease in the average length of telomeres compared to the brain, kidney, lung and liver. [40]. A similar state of telomeres was found in coronary artery endothelial cells, where the T/C ratio (ratio of telomere length to centromeres) was reduced depending on the age of the donor [41]. At the same time, the reduction of the end sections of chromosomes is unlikely to be associated with cell division, as described above.

As for the age-dependent diseases, there is a correlation between the length of telomeres and the presence of one or another heart disease. For example, the risks of coronary heart disease are associated with telomeres shorter than 200 base pairs [42]. The mechanism of a possible causal link between short telomeres and ischemic disease has not been fully elucidated, but shorter telomeres are positively associated with the rapid formation of plaques observed in atherosclerosis and marked atherogenesis [42]. Indeed, direct measurement of telomere length in coronary endothelial cells supports the concept that telomere shortening in coronary endothelial cells with aging can contribute to the development of coronary endothelial dysfunction and the development of coronary heart disease in humans [41].

Dilated cardiomyopathy (DC) is characterized by an increase in cardiac ventricular volumes, thinning ventricular wall thickness, hypertrophy and impairment of cardiac function [43,44]. In an aging heart with DC, the forced entry of primitive cells, which express stem cell surface antigen c-kit, into irreversible quiescent state was identified by the expression of cell cycle inhibitor p16^INK4a^ and by very short telomeres [45].

Telomeric shortening in CMs with age can be explained by the launch of miRNA-34a synthesis, the target of which is phosphatase 1 of the nuclear targeting subunit (PNUTS), which is involved in the maintenance of telomeres by interacting with TRF2. TRF2, together with PNUTS, is also involved in regulating the response to DNA damage and inhibiting the phosphorylation of Chk2 (checkpoint kinase), leading to apoptosis. Increased expression of PNUTS inhibits telomere depletion without telomerase involvement [46]. However, there are probably other molecular pathways that result in a reduction in telomere length. They can be associated, for example, with oxidative stress, leading to the accumulation of DNA damage in telomeric regions.

To determine the possible role of telomeres and telomerase in cardiac aging, mice with telomere- induced dysfunction were examined by knockout on telomerase subunit genes. Mice of the fifth generation (G5) with a *Terc* gene knockout (*Terc*^-/-^) suffered from severe left ventricular insufficiency and DC. Compared to WT mice, the masses of the heart and left ventricle were significantly reduced in G5 mice. Despite the decrease in heart weight in G5, hypertrophy was demonstrated, which was accompanied by a decrease in the number of CMs [47]. The phenotype was also characteristic for G4 *Tert*^-/-^ mice: a decrease in body weight and endurance and an increase in free fatty acids and mitochondrial dysfunction. Mitochondrial dysfunction manifested as inhibition of PGC-1α and PGC-1β, key metabolic regulators. This led to a decrease of gluconeogenesis, a reduction in ATP synthesis, cardiomyopathy, and increased oxidative stress [48], which is a sign of tissue aging [49].

The tumor suppressor protein p53 is an important mediator of telomere dysfunction. An increase in this protein is observed in *Terc*^-/-^ mice when telomeres reach a critically short length. These results are consistent with the notion that telomere loss in mice activates p53, which modulates both apoptosis and growth arrest [47]. In addition to these functions, the p53 protein is a link between telomere length and mitochondrial function: an increase in its synthesis leads to inhibition of the promoters *PGC-1α* and *PGC-1β* [48]. Thus, dysfunction of telomeres leads to premature aging of the heart, which manifests itself in the form of diseases dependent on age.

As described previously [50], premature aging of the cardiovascular system is induced by metabolic stress, obesity, hypertension, insulin resistance and type 2 diabetes [51,52,53,54]. Additionally, there is evidence that autophagy is important for longevity and health [55], and a change in autophagy contributes to heart aging [56,57,58]. Although it has been shown that autophagy inducers have a beneficial effect on life expectancy and slow down the aging of the cardiovascular system [56], there is still a contradiction between the protective and harmful effects of autophagy induction on aging [59]. There are many aging treatment options based on telomerase activation, NO modulation, antioxidants, PARP inhibition, senolytic therapy, plasma membrane redox system (PMRS) activators and stem cell therapy [55,60].

There are reports about the use of telomerase as a therapeutic tissue-specific target for diseases of the cardiovascular system [61,62]. Telomerase can be used to treat coronary heart disease due to protection against ROS [63,64]. With ischemia reperfusion injury, telomerase deficiency leads to heart failure [65]. Telomere depletion is a characteristic sign of cardiac hypertrophy. Shortening of telomeres in CMs is a marker of heart failure in humans, and shorter telomere length in CMs usually correlate with a reduced ejection fraction [66].

## 8. Heart Regeneration 

As we stated above, the regenerative potential of CMs, as well as telomerase activity, decreases in mammalian hearts shortly after birth [13].

In newborn mice (P1), in response to injury, accelerated differentiation of CM occurs [67,68]. Following the P1 period, the regenerative potential is quickly lost, and a similar injury on P7 leads to fibrosis instead of regeneration [67]. Tetraploidy, binucleation, diminished telomerase activity and telomere shortening during this period can be causative of a loss of proliferative potential of CMs in response to injury [13]. A recent study showed that co-cultivation of mononucleated and bi/multinucleated CMs from adult and newborn animals, respectively, led to the de-differentiation and proliferation of not only mononucleated, but also bi/multinucleated CMs, although to a lesser extent [69].

Telomerase expression in the mammalian heart was investigated using transgenic mice expressing green fluorescence protein (GFP) driven by the promoter for murine telomerase reverse transcriptase (mTert), which is a necessary and rate-limiting component of telomerase [19]. Local proliferation of *mTert-GFP*-expressing cells in the adult heart suggests the existence of a subpopulation of mTERT-positive cells that display a phenotype similar to stem cells. This observation is supported by the expression of the heart-specific transcription factors NKX2.5 and GATA4 in these cells, which are necessary for the differentiation into CM lineage. These factors are described as distinctive features of the native stem cells of the heart. A marked local increase in their number in response to trauma in the adult heart indicates their role in regeneration [19]. A similar increase in the number of stem-like cells with the surface antigen Sca-1 and c- kit and their proliferation was observed during a heart attack, which was accompanied by an increase in telomerase activity [70]. Telomerase delays growth arrest, aging and prevents cell death. It may also be involved in the fight against mechanical and oxidative stress [71], which increases with a concomitant increase in ROS during necrosis and inflammation [72].

Conditions of hypoxia can reduce the oxidative stress after induced myocardial infarction. This leads to an increase in proliferation, which further helps to reduce the area of fibrosis and improves systolic function [73]. In addition to these data, the possibility was found of increasing the regenerative potential of hearts at one week of postnatal development of mice after administration of the antioxidant *N*-acetyl-*L*-cysteine [21].

Cardiac muscle regeneration after an injury is complicated by an “irreversible” exit of CMs from the cell cycle. A forced expression of *Tert* in the cardiac muscle in mice is sufficient to restore telomerase activity and telomere length. This, in turn, can delay the exit from the cell cycle in the cardiac muscle, cause hypertrophy in postmitotic cells and contribute to CM survival [14].

To elucidate a role of telomerase in cardiac regeneration, *Tert* was overexpressed in mouse hearts by adeno-associated viral delivery [14,20]. Mice were subjected to experimental myocardial infarction (MI). Upon MI, *Tert*-expressing hearts showed attenuated cardiac dilation, improved ventricular function and smaller infarct scars concomitant with increased survival by 17% compared with controls. Cardiac transcriptome analysis revealed an increase of epidermal growth factor receptor (EGF) in *Tert*-expressing hearts. Signaling through EGF is cardio-protective, emphasizing defensive function of Tert. Tert therapy also leads to activation of pathways associated with extracellular matrix remodeling (an increase in serum MMP-9 and TGF-b). TGF-b has a pleiotropic effect on almost all cell types involved in the repair and heart remodeling after injury. Long-term activation of these genes may also be a consequence of enhanced heart regeneration, which requires matrix remodeling to integrate new CMs. Indeed, the CM can re-enter the cell cycle after injury. Expression of Ki-67 and the presence of phosphorylated forms of histone H3 (proteins, the maximum expression of which coincides with mitosis) was found to increase the number of proliferating CMs near the infarct zone in the Tert-treated group. According to these results, it is plausible that activation of Tert may assist cardiac regeneration [13].

After injury, sufficient telomere length is required for proliferation. In P1 G3 *Terc*-null newborn mice, the proliferative capacity is lower than that of WT controls. Instead of increasing proliferation, CMs of G3 *Terc*^-/-^ mice grow hypertrophic. The aggravation of telomere shortening after cryogenic damage at P1 in the heart of G3 mice causes an increase of p21 levels compared to WTs, which indicates the activation of the DNA damage response. This leads to the cessation of the cell cycle. A reliable proliferative response was observed in CMs of p21^-/-^ mice that were seven days old. This is the age at which the CMs of WT mice lose the ability to divide after injury. This observation further emphasizes the participation of telomerase inhibition through the expression of p21 in stopping cell cycle and inhibition of the regeneration reaction after injury in the early postnatal period [13].

The proliferative ability is obviously associated with increased telomerase activity relative to mammals; therefore, the *D. rerio* model is interesting from the point of view of the possibility of regeneration of CMs.

In a recent study, it was shown that cryogenic damage to the heart leads to an increase in telomerase activity in WT zebrafish, which was associated with an increase in *tert* gene expression. To determine the role of telomeres in regeneration, damage to the heart was performed with *tert*^-/-^ and WT *D. rerio*. It was shown that the cardiac output was not restored, and the area of damage did not decrease in *tert*^-/-^ fish, which indicates the need for telomerase during regeneration. However, the level of the inflammatory response, an important process for regeneration, was the same for WT and *tert*^-/-^ fish. Similarly, dedifferentiation of CMs in response to an injury occurs normally in the absence of telomerase. The length of telomeres increases only in WT CMs during regeneration, and this is characteristic of both actively proliferating cells and nondividing cells. Therefore, we can conclude that telomere elongation is important for CM regeneration [27].

Induction of polyploidy in the heart of zebrafish leads to a loss of regenerative potential after injury [29]. Consequently, for recovery of the myocardium, diploid CMs are required, the number of which decreases in the mammalian heart with age as a result of telomere depletion [13].

## 9. Conclusions

Thus, telomerase activity and telomere length are not only markers of cellular aging of CMs, but they also make a significant contribution to the development of age-related diseases. Further study of the influence of various factors on the telomerase complex and telomeric regions of chromosomes can contribute to a better understanding of the processes of telomere length changes that occur in cardiac tissue throughout life. 

Studies of the regenerative capacity of the hearts of mammals and other vertebrates can also help in the formation of new approaches in the field of regenerative medicine for the treatment of such serious diseases as, for example, myocardial infarction and heart failure.
